# Novel Pathologic Factors for Risk Stratification of Gastric “Indefinite for Dysplasia” Lesions

**DOI:** 10.1155/2020/9460681

**Published:** 2020-09-29

**Authors:** Kwangil Yim, Jung Ha Shin, Jinyoung Yoo

**Affiliations:** ^1^Department of Hospital Pathology, Uijeongbu St. Mary's Hospital, College of Medicine, The Catholic University of Korea, Seoul, Republic of Korea; ^2^Department of Hospital Pathology, Eunpyeong St. Mary's Hospital, College of Medicine, The Catholic University of Korea, Seoul, Republic of Korea; ^3^Department of Hospital Pathology, St. Vincent's Hospital, College of Medicine, The Catholic University of Korea, Seoul, Republic of Korea

## Abstract

**Methods:**

In total, 123 IND cases with final diagnoses of cancer (29.3%), high-grade dysplasia (6.5%), low-grade dysplasia (11.4%), and nonneoplasm (52.8%) were randomly divided into test set (*n* = 27) and validation set (*n* = 96). By the image analysis, size, pleomorphism, hyperchromasia, irregularity of nuclei, and ratios of structural atypia area (SAA) to total IND area were measured in the test set. Using the validation set, consensus meetings were held for the evaluation of pathologic factors that predict the final diagnosis.

**Results:**

By image analysis, the only ratio of SAA to total IND area was associated with the final diagnosis (*p* < 0.001). In the consensus meeting for validation, the nuclear factors, except loss of nuclear polarity (*p* = 0.004–0.026), could not predict the final diagnosis. Conversely, most structural factors could predict the final diagnosis. In particular, SAA > 25% was the most powerful predictive factor. We proposed criteria of risk stratification by using SAA > 25%, loss of surface maturation (LOSM), and loss of nuclear polarity (LONP) (Malignancy rate; Category 0: SAA ≤ 25% without LOSM and LONP; 0%, Category 1: SAA ≤ 25% with any of LOSM or LONP; 15.2%–16.7%, Category 2: SAA > 25% without LOSM and LONP; 44.4%–50.0%, Category 3: SAA > 25% with any of LOSM or LONP 54.5%–55.6%).

**Conclusions:**

Structural atypia was more helpful than nuclear atypia and SAA > 25% was the most powerful predictor for the diagnosis of INDs of the stomach. We propose shortening the follow-up period to six months for Category 1, endoscopic resection for Category 2 and 3, postresection follow-up periods of one year for Category 2, and six months for Category 3.

## 1. Introduction

Pathologic evaluations are the gold standard for the diagnosis of gastric cancer; however, it is not always possible to distinguish between malignancy and benign conditions when architectural distortion or nuclear atypia is present [1–3]. On histomorphologic study, atypical regenerative changes are occasionally indistinguishable from malignancy [4, 5]. The international consensus meeting held in Vienna suggested to designating this indefinite histology as a distinct category, i.e., indefinite for dysplasia (IND) [6, 7].

With regard to the management of the IND, no definite criteria for selecting follow-up methods have been established. Although current guidelines advise follow-up biopsy for IND areas, a few endoscopists insist that endoscopic resection (ER) be used as a follow-up method because malignancy rates in IND areas are high (22.6%–75.0%) [8–12].

In a given stomach tissue sample, reactive changes of the stomach mucosa comprise cytomorphologic changes such as enlarged nuclei, pleomorphism, hyperchromasia, or vesicular changes of nuclei, and these reactive changes might not be associated with neoplastic progress. In contrast, architectural features such as loss of surface maturation (LOSM), margination, glandular cribriform patterns, glandular branching/budding, glandular arrangement, and glandular crowding are relatively more associated with tumorous conditions [4, 13].

In addition to these histomorphologic interpretations, which are qualitative and subjective, quantification of each pathologic factor may facilitate subclassification of IND areas when the cytomorphologic factors do not clearly distinguish neoplasms from reactive changes [14].

With regard to IND follow-up methods, ER has been recently suggested [8–12]. Only a few studies have investigated IND to attempt establishing the criteria of IND follow-up methods and have mostly focused on the clinical factors suggesting malignancy [8–12] or been studied in Barrett's esophagus [1, 2]. The aims of this study were to investigate and quantify pathologic factors for predicting carcinoma in IND through the use of image analysis and thereby facilitate the subclassification of IND conditions and determine the proper follow-up methods.

## 2. Materials and Methods

### 2.1. Patients

We enrolled cases with IND by endoscopic forceps biopsy at the Seoul St. Mary's Hospital (*n* = 137) and St. Vincent's Hospital (*n* = 115) from August 2008 to December 2015. The inclusion criteria for IND lesions were based on the classification of the Japanese Gastric Cancer Association [15]. Patients lost to follow-up after the first biopsy were excluded (St. Mary's Hospital (*n* = 70) and St. Vincent's Hospital (*n* = 59)). A total of 123 cases (St. Mary's Hospital (*n* = 67) and St. Vincent's Hospital (*n* = 56)) were randomly divided into test (*n* = 27) and validation (*n* = 96) sets. The final diagnosis of nonneoplasm was established if three consecutive samples obtained from the same IND area demonstrated no neoplasms or if no detectable lesions were found in endoscopic or radiologic examination for more than 1 year. The study was approved by our institutional review board (XC17RAMI0005K).

### 2.2. Image Analysis

**Figure 1:**
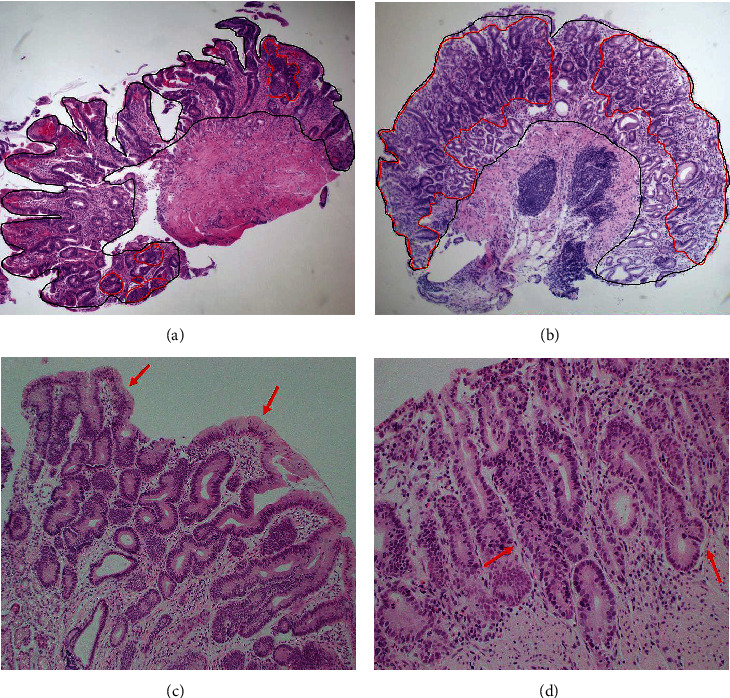
Representative images of structural atypia area (red-lined inner area) and area with total indefinite for dysplasia (black-lined inner area) In the red-lined inner area, one or more of the following are seen: variable glandular size, irregular glandular arrangement, glandular branching/budding, and glandular cribriform. In the black-lined inner area, one or more of structural or nuclear atypia are visible. (a) Structural atypia was present in 6.18% of the area, with a final diagnosis of nonneoplasm (hematoxylin and eosin; ×40 magnification). (b) The area ratio of the structural atypia was 61.1%, with a final diagnosis of carcinoma (hematoxylin and eosin; ×40 magnification). (c) A representative image of an area of nuclear atypia without structural atypia. Loss of surface maturation (arrow), nuclear enlargements, and hyperchromasia are visible (hematoxylin and eosin; ×100 magnification). (d) A representative image of an area of structural atypia. Glandular variation in size, irregular arrangement, and branching/budding (arrow) are visible (hematoxylin and eosin; ×200 magnification).

We evaluated 20 representative nuclei per each sample of the test set (*n* = 27) in IND areas using Image J (National Institutes of Health, Bethesda, United States). The average areas of the nuclei were measured to evaluate mean nuclear areas for nuclear enlargement, standard deviation of nuclear areas for pleomorphism, brightness ratio of tumor to lymphocytes for hyperchromasia, and Feret diameters for nuclear irregularity. The area ratios of structural atypia to IND were calculated to quantify structural atypia. The structural atypia area (SAA) was defined as areas with ≥1 of the following: variable glandular size, irregular glandular arrangement, glandular branching/budding, and glandular cribriform patterns (Figure 1) [16]. The area of IND was defined as an area with any well-known structural anomalies (LOSM, margination, glandular cribriform patterns, glandular branching/budding, glandular arrangement, and glandular crowding) or nuclear atypia (loss of nuclear polarity (LONP), nuclear pseudostratification in more than half, nuclear pleomorphism, nuclear hyperchromasia, and prominent nucleoli) [16]. These measurements were discussed among three pathologists (K Yim, JH Shin, and J Yoo). Receiver operating characteristic (ROC) curves were plotted to determine the cut-off values in the SAA to IND that could predict neoplasm, above high-grade dysplasia (HGD), or carcinoma. The sum of the sensitivity and specificity was obtained, and cut-off values were determined using the maximum sum.

### 2.3. Consensus Meeting to Establish Criteria and Validation

We discussed and confirmed well-known pathologic factors [16] to differentiate between nonneoplasm and neoplasm, below LGD and above HGD, and noncarcinoma and carcinoma in the test set (*n* = 27) and to include a few other factors (neutrophils, ulcer, and intestinal metaplasia) that may affect interpretation. Each of these factors was considered as positive when definitely present in the cases.

These factors were subsequently analyzed and determined in the validation set (*n* = 96) by two pathologists (K Yim and JH Shin). Whether the SAA ratio above the cut-off value determined by image analysis could predict the final diagnosis was also evaluated.

### 2.4. Data and Statistical Analysis

Detailed information, including age and sex, size, location, and shape of the lesions and other potential risk factors, was obtained retrospectively from medical records. The surface nodularity or spontaneous bleeding could not be obtained because it was not recorded in the medical records. Continuous data were compared using independent *t*-tests, and categorical variables were tested using Pearson's *χ*^2^ method or Fisher's exact tests. The criterion for statistical significance was *p* < 0.05. ROC curves were plotted, and the maximum sum of sensitivity and specificity was defined as the cut-off point. Cohen's Kappa was calculated to compare the results of the two pathologists. For multivariate analysis, ROC curves were obtained for each pathologic parameter with crude factors, and differences in area under curve (dAUC) to crude factors were used to compare the predictive power of each pathologic factor. Three effectible factors (neutrophils, ulcer, and intestinal metaplasia) were used as crude factors. Analyses were performed using SPSS (version 18.0; SPSS, Chicago, IL, USA).

## 3. Results

### 3.1. Final Diagnoses of INDs

**Figure 2:**
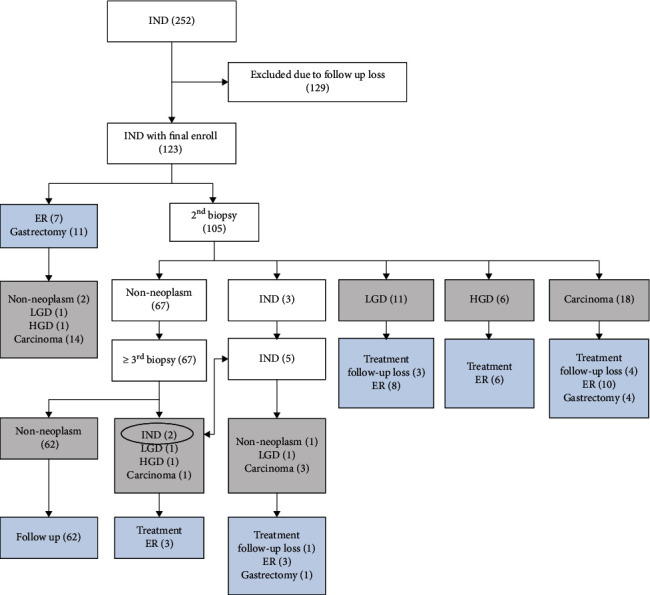
Flow chart of study inclusion and exclusion criteria and enrolment of 123 lesions with indefinite for dysplasia. ER: endoscopic resection; HGD: high-grade dysplasia; IND: indefinite for dysplasia; LGD: low-grade dysplasia.

The enrolled patients (*n* = 123) underwent follow-up biopsy (*n* = 105, 85.4%), ER (*n* = 7, 5.7%), or gastrectomy (*n* = 11, 8.9%). The reason for gastrectomy after a diagnosis of IND in six patients was the presence of coexisting lesions in distant sites of the stomach (The final diagnoses were carcinomas in four patients and nonneoplasms in two). Radiological examination revealed overt malignant findings in the four patients with a final diagnosis of carcinoma. One of those eleven patients exhibited marked fibrosis and ulcer; therefore, obtaining an adequate sample by biopsy was difficult, and, furthermore, ER was not available. The patient himself wanted surgery since he did not want to worry about cancer (The final diagnosis was a carcinoma). After complete workup, the final pathologic diagnoses included cancer (*n* = 36, 29.3%), HGD (*n* = 8, 6.5%), LGD (*n* = 14, 11.4%), and nonneoplasms (*n* = 65, 52.8%). Among the cases with the final diagnosis of carcinoma, 19 (52.8%) were determined by biopsies, 8 (22.2%) by ER, and 9 (25.0%) by gastrectomy. Although 67 cases showed nonneoplasm at second follow-up biopsies, after a series of follow-up biopsies, two cases showed persistent INDs (final diagnoses were carcinomas), one showed LGD, one showed HGD, and one showed cancer. The final diagnoses of persistent INDs were one nonneoplasm, one LGD, and three carcinomas (Figure 2).

### 3.2. Diagnostic Delays and Prognostic Impacts

The average delay in diagnosis and treatment of IND was 239.2 ± 362.5 days (3–2157 days) and 217.8 ± 294.5 days (6–1498 days). Time taken for definite diagnosis was <2 weeks for 13 cases (10.6%), ≥2 weeks for 110 cases (89.4%), ≥2 months for 79 cases (64.2%), ≥6 months for 43 cases (34.1%), and ≥1 year for 27 cases (22.0%) (Table 1).

There were more persistent INDs or negative in the second biopsy at the same lesion in those with delayed diagnosis (2 weeks: *p* = 0.598, 2 months: *p* = 0.257, 6 months: *p* = 0.021, 1 year: *p* = 0.013). However, there were no significant differences in the final pathologic stage (Table 1). Furthermore, there were eight cases with repeated IND or negative in the second biopsy, one was nonneoplasm (12.5%), three were LGDs (37.5%), and three were carcinomas with mucosal invasion (37.5%), and one with carcinoma accompanied by submucosal invasion (12.5%) (data not shown).

### 3.3. Endoscopic Findings and Clinical Factors

Of the 123 INDs at initial biopsy, 58 (47.2%) patients were finally diagnosed as having neoplasm. Endoscopic findings showed that the mean size of neoplastic lesions was smaller than that of nonneoplasms (2.86 ± 2.09 vs. 3.00 ± 2.66, *p* = 0.752,) and the mean size of carcinomas was smaller than that of noncarcinomas (2.78 ± 1.79 vs. 3.00 ± 2.62, *p* = 0.465). The nonneoplasm group had a greater percentage of larger lesions ≥ 10 mm than the neoplasm group (6.1% vs. 3.4%, *p* = 0.09). Furthermore, the noncarcinoma group had a greater percentage of larger lesions (≥10 mm) than the carcinoma group (5.7% vs. 2.8%, *p* = 0.09). Also, no significant differences were found for lesion locations, gross types, and number of biopsies among neoplasm vs. nonneoplasm and carcinoma vs. noncarcinoma (Table 2).

### 3.4. Image Analysis

**Figure 3:**
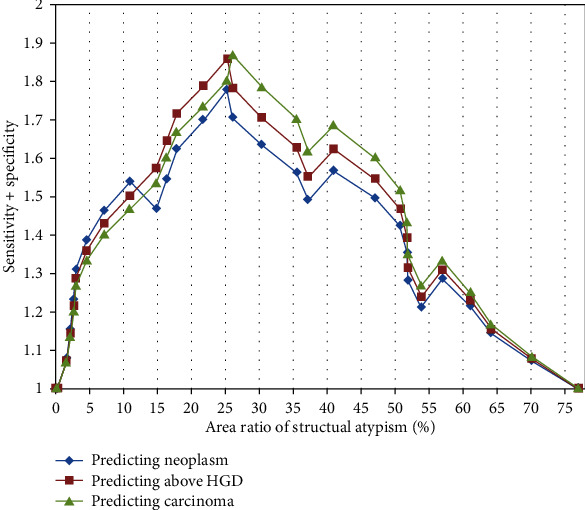
Sum of the sensitivity and specificity for predicting final diagnoses according to area ratio with structural atypia to total indefinite for dysplasia by plotting receiver operating characteristic curves. Cut-off values were defined as 25.3% for neoplasm, 25.3% for above high-grade dysplasia, and 26.1% for carcinoma.

There were no significant differences among all groups for any of the parameters for nuclear atypia (neoplasm vs. nonneoplasm, below LGD vs. above HGD, and noncarcinoma vs. carcinoma) (Table 3). Only area ratios of structural atypia to IND was significantly higher in neoplasm than nonneoplasm (46.4% vs. 15.1%, *p* < 0.001), in above HGD than below LGD (48.9% vs. 15.0%, *p* < 0.001), and in carcinoma than noncarcinoma (50.8% vs. 15.7%, *p* < 0.001) (Table 3). The cut-off values for the area ratio with structural atypia were 25.3% for neoplasm, 25.3% for above HGD, and 26.1% for carcinoma when calculated as the maximum (sensitivity + specificity) point using ROC (Figure 3).

### 3.5. Validation Consensus Meeting

The interpretation results by each pathologist are summarized in Table 4. The factors that both pathologists interpreted as significant predictor for carcinoma were LOSM (*p* = 0.001, *p* = 0.012), glandular cribriform (*p* < 0.001, *p* = 0.001), glandular branching/budding (*p* < 0.001, *p* = 0.001), glandular arrangement (*p* < 0.001, *p* = 0.002), SAA > 25% (*p* < 0.001), and LONP (*p* = 0.004, *p* = 0.026). Cohen's Kappa coefficients were >0.9 for neutrophils, ulcer, and SAA > 25%; 0.4–0.7 for hyperchromasia, cribriform and branching/budding; and 0.7–0.9 for all the other pathologic factors.

### 3.6. Multivariate Analysis of Pathologic Factors for Predicting Final Diagnosis

**Figure 4:**
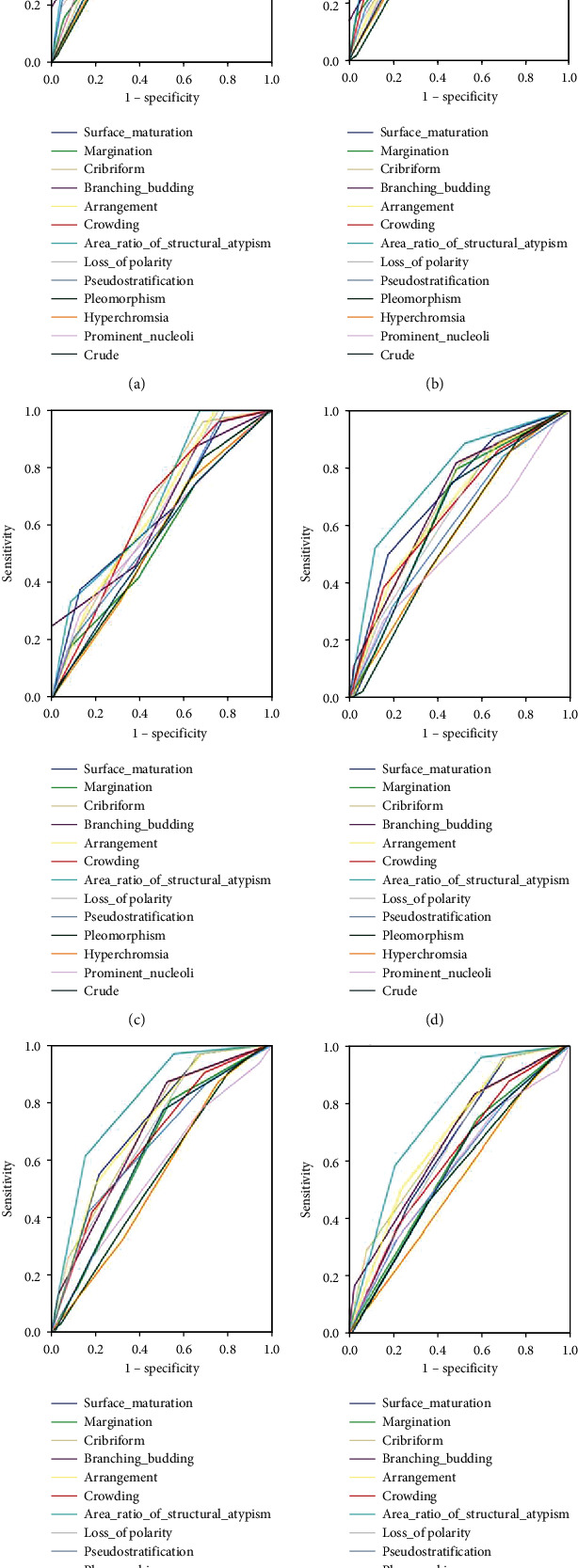
Multivariate analysis of pathologic factors for predicting final diagnosis. Data for the difference in area under the curve are summarized in Table 5.

SAA > 25% was the best predictor for the final diagnosis among the three groups (neoplasm, dAUC = 0.130 and 0.110; above HGD, dAUC = 0.110–0.173 carcinoma, dAUC = 0.135–0.182). LOSM was the second best predictor for the final diagnosis in the neoplasm (dAUC = 0.074–0.083) and above HGD (dAUC = 0.083–0.098) groups. However, in the carcinoma group, pathologist1 interpreted that glandular arrangement (dAUC = 0.115) was a more predictive value than LOSM (dAUC = 0.081), and by pathologist2, glandular cribriform (dAUC = 0.113) showed a more predictive value than LOSM (dAUC = 0.090) (Figure 4 and Table 5).

The risk stratification using SAA > 25%, LOSM, and LONP for predicting carcinoma is summarized in Table 6. The malignancy rates were 0% in Category 0, 15.2%–16.7% in Category 1, 44.4%–50.0% in Category 2, and 54.5%–55.6% in Category 3 (Table 6).

## 4. Discussion

In the present study, 36/123 cases (29.3%) of IND lesions at initial biopsy were finally determined to be gastric cancers, which is similar to the rates observed in previous studies (22.6%–75.0%) [8–12]. Although currently, follow-up biopsy is usually recommended, recently, and ER has been proposed as a follow-up method because of high malignancy rates and diagnostic difficulty of IND [8–12]. Similarly, the diagnostic difficulty of IND in biopsy specimen was observed in the present study. The predictive power of biopsy was relatively low (cancers in biopsy: 52.8%, biopsy as follow-up methods: 85.3%), and persistent IND or false-negative cases (6.5%) were shown.

However, the use of ER as a follow-up method after IND diagnosis remains controversial because most tumors with IND are well-differentiated and slow growing [12, 17, 18]. Similar to previous findings, the present study showed that diagnostic delay did not affect the final pathological stage [12], and most tumors, even with false-negative diagnoses or persistent IND, were well-differentiated tumors that remained in the early gastric cancer stage (Table 2).

There were some attempts to establish guidelines for follow-up methods of INDs. Most previous studies about IND were focused on the clinical features suggesting malignancy [8–12], diagnosis agreements of pathologists [1, 2, 12], INDs of Barrett's esophagus [1, 2], or diagnostic progress by additional immunohistochemistry [1, 2]. Reportedly, ≥10 mm lesion size, depressed lesion, spontaneous bleeding, and surface nodularity on endoscopy were independent risk factors for gastric cancer [10–12]. However, other studies had shown that endoscopic lesion size, gross appearance, location, number of biopsies, and *Helicobacter pylori* infection were not predictive of gastric cancer [8, 9]. In the present study, endoscopic lesion size, gross appearance, location, and number of biopsies were not associated with the final diagnosis.

To the best of our knowledge, pathological analysis for IND in gastric specimen had not been attempted until Kwon et al. [12] reported that distorted architecture, i.e., increased glandular crowding and irregular glandular arrangements, was strongly associated with carcinoma. The present study evaluated and quantified each pathologic factor that could further classify INDs in gastric biopsy specimen. As a result, we established a novel SAA > 25% as a quantitative pathologic factor in this study. This novel factor integrated variable glandular size, irregular glandular arrangement, glandular branching/budding, and glandular cribriform by measuring areas with ≥1 of the above, and it was the best predictor for final diagnosis (dAUC = 0.135–0.182 for predicting carcinoma, Figure 4 and Table 5) and showed high reproducibility (Cohen′s Kappa coefficient = 0.956). Therefore, the quantification of pathologic factors, while at a basal level, could be helpful for an accurate diagnosis. SAA > 25% could not integrate LOSM, margination, and all factors regarding nuclear atypia because these factors were difficult to quantify by area measurement.

Nuclear atypia could not predict the final diagnosis, except for LONP (*p* = 0.004–0.026, Cohen′s Kappa = 0.869). Reactive changes, including inflammation or ulcers, may lead to enlarged nuclei and pleomorphism of nuclei nuclear hyperchromasia or vesicular changes [4, 13]. In contrast, most pathologic factors associated with structural atypia showed good predictability for the final diagnosis that strongly correlated between the two pathologists (Cohen′s Kappa = 0.682–0.855, Table 4). This confirmed that changes in the structure were relatively preserved in the reactive settings, such as inflammations or ulcers [4, 13].

## 5. Conclusions

In conclusion, we propose criteria for the risk stratification of INDs, as summarized in Table 6. The criteria were based on the following: (1) Only factors with statistical significance by both pathologists were included. (2) The predictive pathologic factors could be grouped into SAA > 25%, LOSM, and LONP. (3) SAA > 25% was the best predictor for diagnosis. (4) The ability to detect cancer was focused than premalignant lesions. Because most tumors in IND areas are well-differentiated and slow growing, premalignant lesions can be monitored with follow-up biopsy [12, 17, 18]. According to the criteria, Category 0 had no cancer risk; therefore, routine annual follow-up might be sufficient. Category 1 had a cancer risk of 15.2%–16.7%; hence, we suggest follow-up and repeat biopsy after 6 months. Because Category 2 (44.4%–50.0%) and Category 3 (54.5%–55.6%) had relative high cancer risk, ER should be recommended. In patients who have undergone ER, we suggest follow-up after 1 year for Category 2 and after 6 months for Category 3 disease [19, 20]. (Table 6).

Our study has some limitations. Interobserver variation between pathologists was not fully overcome. Furthermore, this can be aggravated among the pathologists of different training experiences. Also, this was a retrospective study and lacked strictly regulated, periodical follow-up and endoscopic data, such as nodularity or spontaneous bleeding, which were factors predictive of carcinoma according to a previous study [12]. Future large prospective research is required to confirm our results. Nevertheless, our study attempted to quantify pathologic factors, and we showed that this could work well in difficult diagnostic areas, such as IND. Adding SAA > 25% to pathology reports may be helpful for the treatment of INDs. Also, our institute has proposed criteria for risk stratification for INDs with distinctly different malignancy rates, and this may enable subclassification of INDs into low- and high-risk groups with different follow-up or treatment methods.

## Figures and Tables

**Table 1:** The diagnostic delays and clinicopathological correlation.

	Early <2 weeks (*n* = 13)	Delayed ≥2 weeks (*n* = 110)	*p*	Early <2 months (*n* = 44)	Delayed ≥2 months (*n* = 79)	*p*	Early <6 months (*n* = 80)	Delayed ≥6 months (*n* = 43)	*p*	Early <1 year (*n* = 96)	Delayed ≥1 year (*n* = 27)	*p*
Repeated IND/negative in 2^nd^ biopsy												
No	13	102	0.598	43	72	0.257	78	37	0.021	93	22	0.013
Yes	0	8		1	7		2	6		3	5	
Stage												
≤pT1a	11	103	0.242	39	75	0.279	75	39	0.718	90	24	0.41
≥pT1b	2	7		5	4		5	4		6	3	

IND: indefinite for dysplasia.

**Table 2:** Clinical and endoscopic features according to final diagnosis.

	Nonneoplasm (*n* = 65)	Neoplasm (*n* = 58)	*p*	Noncarcinoma (*n* = 87)	Carcinoma (*n* = 36)	*p*
Age (years old)	64.8 ± 12.2	67.8 ± 8.97	0.126	65.2 ± 11.5	68.8 ± 8.64	0.092
Sex						
Male	49	40	0.427	63	26	0.574
Female	16	18		24	10	
Endoscopic findings						
Mean size (mm)	3.00 ± 2.66	2.86 ± 2.09	0.752	3.00 ± 2.62	2.78 ± 1.79	0.465
<4 mm	59	51	0.09	78	32	0.09
≥4 mm and <10 mm	2	5		4	3	
≥10 mm	4	2		5	1	
Location						
Lower third	41	31	0.09	50	22	0.094
Mid third	22	25		35	12	
Upper third	2	2		2	2	
Gross type						
Type I	3	8	0.09	4	7	0.101
Type IIa	8	4		10	2	
Type IIb	9	11		14	6	
Type IIc	41	29		54	16	
Type III	4	6		5	5	
Number of biopsies	2.02 ± 1.42	2.17 ± 1.48	0.549	1.98 ± 1.27	2.36 ± 1.79	0.18

Data are presented number or mean with standard deviation (mm).

**Table 3:** The Results of image analysis for pathologic factors associated with nuclear and structural atypia.

		Nonneoplasm	Neoplasm	*p*	Below LGD	Above HGD	*p*	Noncarcinoma	Carcinoma	*p*
		(*n* = 13)	(*n* = 14)		(*n* = 14)	(*n* = 13)		(*n* = 15)	(*n* = 12)	
Nuclear	Size (*μ*m^2^)	16.3 ± 3.4	18.6 ± 5.0	0.186	16.3 ± 3.3	18.7 ± 5.1	0.156	16.6 ± 3.3	18.6 ± 5.3	0.237
	Pleomorphism (*μ*m^2^)	5.6 ± 2.1	5.5 ± 1.7	0.806	5.5 ± 2.0	5.6 ± 1.7	0.895	5.6 ± 2.0	5.5 ± 1.8	0.957
	Hyperchromasia (%)	79.8 ± 8.0	81.0 ± 7.9	0.689	79.4 ± 7.9	81.6 ± 7.9	0.48	79.0 ± 7.7	82.2 ± 7.9	0.302
	Irregularity (*μ*m)	3.0 ± 0.29	3.2 ± 0.4	0.137	3.0 ± 0.29	3.2 ± 0.41	0.969	3.0 ± 0.31	3.2 ± 0.42	0.294
Structure	Area ratio (%)	15.1 ± 16.4	46.4 ± 17.3	<0.001	15.0 ± 15.7	48.9 ± 15.1	<0.001	15.7 ± 15.4	50.8 ± 14.0	<0.001

Data are presented mean with standard deviation (%, *μ*m and *μ*m^2^). LGD: low-grade dysplasia; HGD: high-grade dysplasia.

**Table 4:** Interpretation of results of pathologic factors for predicting carcinoma by two pathologists.

			Pathologist1			Pathologist2			Cohen's Kappa coefficients
			Noncarcinoma (*n* = 72)	Carcinoma (*n* = 24)	*p*	Noncarcinoma (*n* = 72)	Carcinoma (*n* = 24)	*p*	
Effectible factors	Neutrophils	≤mild	34	10	0.408	1	0	0.75	0.959
		≥moderate	38	14		74	24		
	Ulcer	Absent	41	17	0.168	43	17	0.234	0.956
		Present	31	7		29	7		
	Intestinal metaplasia	<50%	65	24	0.124	68	24	0.31	0.712
		≥50%	7	0		4	0		

Structural atypia	Loss of surface maturation	Absent	37	3	0.001	42	7	0.012	0.813
		Present	35	21		30	17		
	Margination	Absent	61	19	0.364	62	19	0.304	0.731
		Present	11	5		10	5		
	Cribriform	Absent	58	9	<0.001	65	14	0.001	0.664
		Present	14	15		7	10		
	Branching/budding	Absent	67	13	<0.001	70	17	0.001	0.682
		Present	5	11		2	7		
	Arrangement	Regular	40	3	<0.001	44	6	0.002	0.855
		Irregular	32	21		28	18		
	Crowding	Absent	43	5	0.001	45	11	0.116	0.833
		Present	29	19		27	13		
	SAA > 25%	No	56	5	<0.001	55	4	<0.001	0.956
		Yes	16	19		17	20		

Nuclear atypia	Loss of nuclear polarity	Absent	48	8	0.004	51	11	0.026	0.869
		Present	24	16		21	13		
	Pseudostratification	<half	39	7	0.029	43	13	0.403	0.793
		≥half	33	24		29	11		
	Pleomorphism	Absent	65	20	0.279	67	21	0.318	0.825
		Present	7	4		5	3		
	Hyperchromasia or vesicular	Absent	2	0	0.561	4	2	0.469	0.484
		Present	70	24		68	22		
	Prominent nucleoli	Absent	20	3	0.104	23	6	0.356	0.843
		Present	52	21		49	18		

SAA: structural atypia area.

**Table 5:** Multivariate analysis of pathologic factors for predicting final diagnosis.

		Predicting neoplasm		Predicting above HGD		Predicting carcinoma	
		Pathologist1	Pathologist2	Pathologist1	Pathologist2	Pathologist1	Pathologist2
		dAUC	dAUC	dAUC	dAUC	dAUC	dAUC
Structural atypia	Loss of surface maturation	0.074	0.083	0.098	0.083	0.081	0.090
	Margination	0.012	0.019	0.000	0.019	0.013	0.000
	Cribriform	0.041	0.051	0.074	0.051	0.103	0.113
	Branching/budding	0.050	0.073	0.066	0.073	0.095	0.086
	Arrangement	0.027	0.038	0.094	0.038	0.115	0.102
	Crowding	0.021	0.065	0.039	0.065	0.042	0.095
	SAA > 25%	0.130	0.110	0.173	0.110	0.182	0.135

Nuclear atypia	Loss of nuclear polarity	0.007	0.024	0.064	0.024	0.080	0.083
	Pseudostratification	−0.036	0.019	0.027	0.019	0.005	0.057
	Pleomorphism	−0.068	−0.030	−0.079	−0.030	−0.016	0.010
	Hyperchromasia	−0.062	−0.016	−0.088	−0.016	−0.047	0.000
	Prominent nucleoli	−0.095	−0.036	−0.068	−0.036	0.002	0.041

dAUC: differences in area under curve; HGD: high-grade dysplasia; SAA: structural atypia area.

**Table 6:** Criteria for risk stratification of indefinite for dysplasia and malignancy rates.

SAA > 25%	Loss of surface maturation Loss of nuclear polarity	Category	Malignancy rate
No	None of the above	0	0%
	≥1 of the above	1	15.2–16.7%
Yes	None of the above	2	44.4–50.0%
	≥1 of the above	3	54.5–55.6%

SAA: structural atypia area.

## Data Availability

The data used to support the findings of this study are available from the corresponding author upon request.

## Supplementary Materials

Supplementary 1 Examples of well-known structural and nuclear pathologic factors. (a) Representative image of glandular cribriform pattern, loss of nuclear polarity (arrow), and nuclear pleomorphism. (hematoxylin and eosin; ×200 magnification). (b) Representative image of nuclear pseudostratification in more than half (arrow) and nuclear hyperchromasia. (hematoxylin and eosin; ×400 magnification).

Supplementary 2 Representative images with quantified data by using Image J software. By using wand tool (arrow), we adjust to represent the real nucleus and measure 20 representative nuclei with area, mean brightness, and Feret diameter.
