# Supplementary figures and images for: Novel Pathologic Factors for Risk Stratification of Gastric “Indefinite for Dysplasia” Lesions
Source: Gastroenterol Res Pract. 2020 Sep 29;2020:9460681. doi: 10.1155/2020/9460681 (PMC7542492; doi:10.1155/2020/9460681)

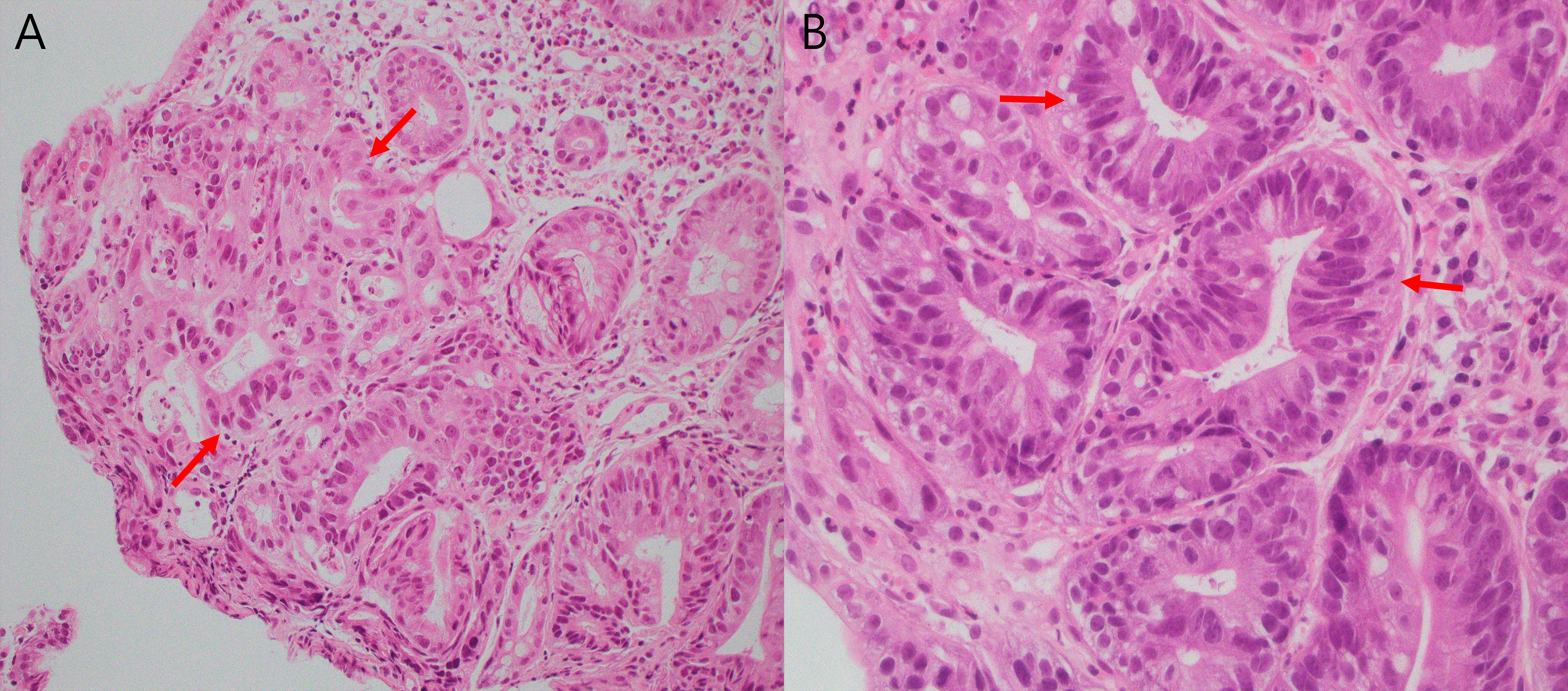

Supplement: Supplementary 1 — Examples of well-known structural and nuclear pathologic factors. (a) Representative image of glandular cribriform pattern, loss of nuclear polarity (arrow), and nuclear pleomorphism. (hematoxylin and eosin; ×200 magnification). (b) Representative image of nuclear pseudostratification in more than half (arrow) and nuclear hyperchromasia. (hematoxylin and eosin; ×400 magnification). [file 9460681.f1.tif]

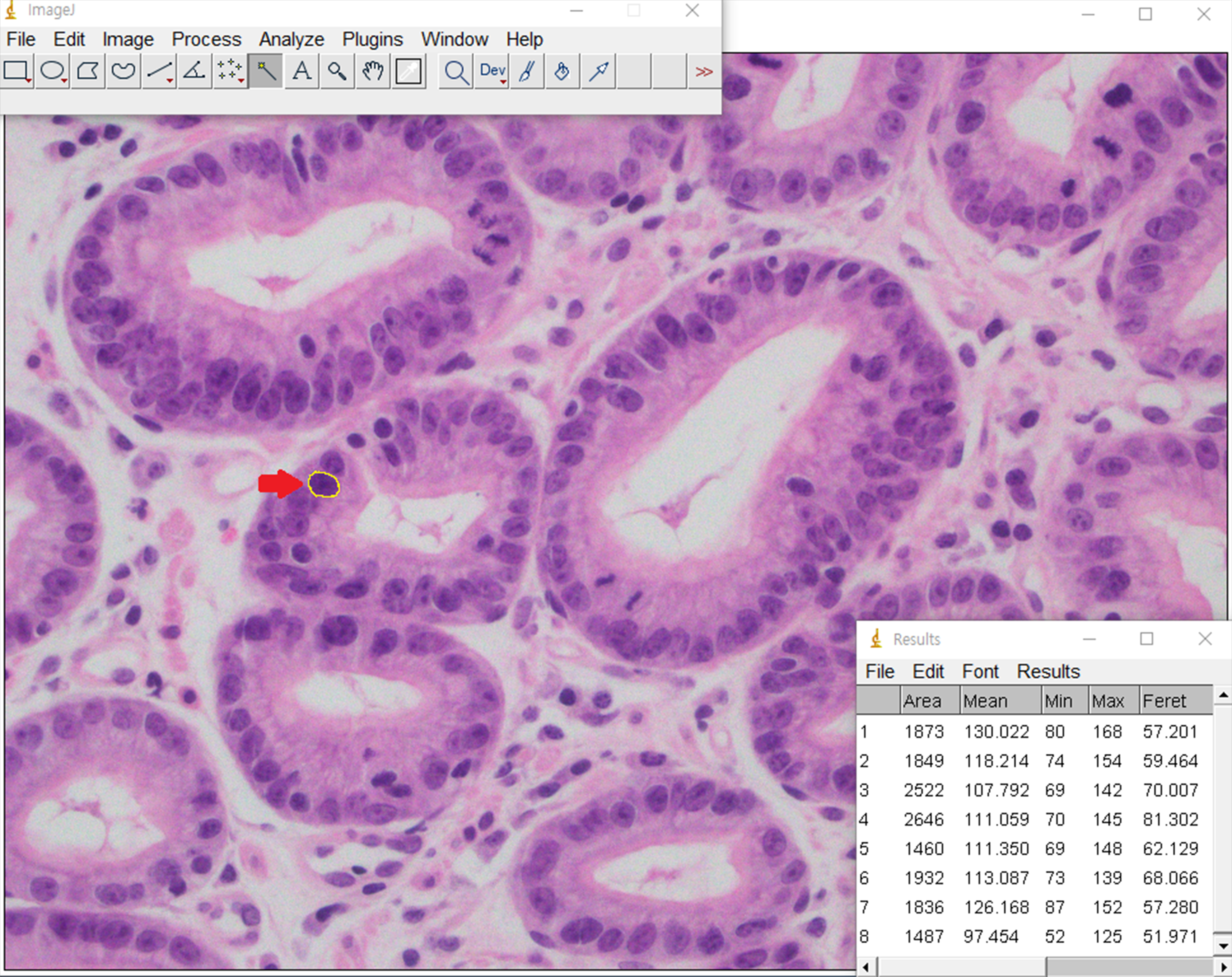

Supplement: Supplementary 2 — Representative images with quantified data by using Image J software. By using wand tool (arrow), we adjust to represent the real nucleus and measure 20 representative nuclei with area, mean brightness, and Feret diameter. [file 9460681.f2.tif]
